# Using an Osteochondral Allograft Procedure for a Large, Unsalvageable Osteochondritis Dissecans Lesion in the Posterior Lateral Femoral Condyle: A Case Report

**DOI:** 10.7759/cureus.90973

**Published:** 2025-08-25

**Authors:** Timothy R Kanne, Jeffrey C McCloud, John R Lusk, Charles W Sanderlin

**Affiliations:** 1 Department of Surgery, Edward Via College of Osteopathic Medicine (VCOM) Auburn, Auburn, USA; 2 Department of Sports Medicine, Valdosta Orthopedic Associates, Valdosta, USA

**Keywords:** lateral femoral condyle, osteochondral allograft, osteochondritis dissecans, posterior lateral femoral condyle, unsalvageable ocd lesion

## Abstract

Osteochondritis dissecans (OCD) is an acquired condition in which articular cartilage and subchondral bone become ischemic and necrotic. While not an uncommon etiology of medial adolescent knee pain, it infrequently forms in the lateral femoral condyle and even less so in the posterior lateral femoral condyle. In this case report, we describe the management of a 13-year-old boy with an OCD lesion in the weight-bearing portion of the posterior lateral femoral condyle. Initially managed nonoperatively, the patient ultimately required an arthroscopic microfracture procedure, followed by an osteochondral allograft (OCA) procedure. This case highlights the application of osteochondral allograft procedures for treating unsalvageable OCD lesions in atypical locations, demonstrating their efficacy in restoring joint function and enabling a return to physical activity.

## Introduction

Osteochondritis dissecans (OCD) is a joint disorder characterized by the separation of a segment of the bone and its adjacent cartilage due to a loss of blood supply. This condition is most common in adolescent men and can lead to pain and impaired joint function [1]. OCD lesions are most commonly found in the knee, but they can also occur in the elbow, ankle, and other joints [2]. Knee OCD lesions occur with an incidence rate of 15.4 per 100,000 men, most commonly in ages 12-19, and occur in the medial femoral condyle in approximately two-thirds of patients and the lateral femoral condyle in approximately one-third of patients [3]. OCD lesions in the lateral femoral condyle deserve careful attention since morphological abnormalities in the lateral femoral condyle are associated with ipsilateral and contralateral anterior cruciate ligament (ACL) injuries, valgus abnormalities, and consequent lateral compartment osteoarthritis [4,5].

The exact cause of OCD is not fully understood. Evidence suggests a multifactorial etiology involving genetics and endocrine disturbances such as vitamin D deficiency, but the best-supported hypothesis involves repetitive trauma or microtrauma to the joint leading to the disruption of the blood supply to the subchondral bone [2,6]. Repetitive trauma to the anterior tibial spine has been linked to medial femoral condyle OCD lesions, as well as having a relatively narrow intercondylar notch, which can cause the impingement of the anterior tibial spine against the medial femoral condyle [7]. Having a relatively distal femoral posterior cruciate ligament (PCL) footprint has also been associated with medial OCD lesions due to repetitive traction loading [2,6]. Lateral femoral condyle OCD lesions are associated with similar morphological abnormalities, such as having a discoid lateral meniscus. Complete discoid menisci are associated with central OCD lesions, while incomplete discoid menisci are associated with peripheral OCD lesions, presumably because central discoid menisci lead to a more central and uniform loading [8]. These abnormal morphologies ultimately result in the death of the bone and the subsequent separation of the affected segment [2].

Patients with OCD lesions typically present with vague, poorly localized joint pain with activity but can also present with an unremarkable knee physical examination. The most common symptom is pain with weight-bearing, but symptoms can vary depending on the size and location of the lesion, as well as its stability [1,2]. Unstable or loose fragments can cause mechanical symptoms such as clicking and locking and indicate advanced disease [1]. Recognizing these symptoms early is critical, as a timely diagnosis can significantly influence treatment outcomes.

The confirmation of OCD lesions is made through imaging studies. Plain radiographs are the initial modality. Preferred views include weight-bearing anteroposterior (AP) and lateral views, as well as a tunnel or notch view, which is an AP view with the knee between 30 and 50 degrees of flexion [1]. The presence of subchondral irregularities can indicate an OCD lesion but can also be a normal variant in patients with open growth plates. Even when accurately detected, radiographs cannot determine lesion stability [2]. For these reasons, magnetic resonance imaging (MRI) is the preferred imaging modality. MRI can characterize the size of the lesion, the status of subchondral bone and cartilage, and the presence of loose bodies [1]. Despite MRI’s high sensitivity in detecting and characterizing OCD lesions, significant discordance exists between MRI and intraoperative findings [9]. For this reason, some recommend that, when lesions warrant surgical treatment, diagnostic arthroscopy should be the first step [6].

The treatment for OCD lesions aims to promote stability while preserving articular cartilage. Treatment options depend primarily on the stability of the lesion and whether the patient’s physes are closed or not. Multiple MRI classification systems of stability exist, but the common features of instability include high T2 signal intensity around the lesion, particularly if it has the same intensity as nearby joint fluid, the disruption of articular cartilage, perilesional cysts larger than 5 mm, and multiple breaks in the subchondral bone [2]. The treatment of stable lesions in patients with open physes includes restricted weight-bearing and immobilization for three to six months, depending on physician preference, which will result in healing without fragmentation in 50%-75% of patients [1,2]. For those with open physes and stable OCD lesions that have failed conservative treatment, both transarticular and retroarticular drilling have been shown to have excellent results. Transarticular drilling can be done with direct visualization during arthroscopy but violates overlying articular cartilage. Retroarticular drilling spares the physis and avoids damaging articular cartilage but is technically challenging and requires fluoroscopy. However, both have good radiographic healing (91% on average of 4.5 months for transarticular and 86% on average of 5.6 months for retroarticular) without complications [10]. Additionally, microfracture surgery is indicated for patients with open physes and stable lesions that are no bigger than 2-3 cm^2^ who have failed conservative treatment and has been shown to yield satisfactory or excellent results in 63% of juvenile patients [2]. For patients with open physes and unstable lesions, treatment should start with diagnostic arthroscopy to directly visualize the lesion and its fragments and to assess how much intact subchondral bone is present. Fixation techniques have been shown to be successful include metal compression screws, absorbable screws and nails, autologous bone sticks, and cell-based cartilage restoration procedures [11-17]. However, these techniques generally require 2-3 mm of intact subchondral bone, and care must be taken not to fracture the fragment being fixated [10].

For patients with closed physes and stable OCD lesions, treatment should only be initiated if they are symptomatic. Conservative management is a reasonable option, but the threshold for moving to surgical treatment is lower than patients with open physes. When surgery is indicated, the preferred procedure depends on whether the lesion is considered salvageable or not. Lesions are considered salvageable and eligible for fixation if the subchondral bone is intact or minimally damaged, if the lesion is in non-weight-bearing areas, if bone fragments can be securely fixed to the bone, and if the overlying articular cartilage is relatively healthy [10]. Otherwise, osteochondral autograft transplantation system (OATS), osteochondral allograft (OCA), or autologous chondrocyte implantation (ACI) may be indicated. OATS is most commonly used for lesions between 0.5 and 3 cm^2^ and has reported satisfactory clinical and radiologic outcomes in 83%-100% of patients [2]. When multiple plugs are required to fill a defect, this procedure is termed a mosaicplasty. Drawbacks include harvest site morbidity, difficulty in matching the exact shape of the lesion, and the limited amount of cartilage that can be harvested [10]. ACI is often used for defects larger than 3 cm^2^ [2]. It consists of harvesting the patient’s cartilage from a non-weight-bearing portion of the knee, growing additional chondrocytes in culture in a laboratory, and implanting them back into the patient’s defect [18]. Although expensive and time-consuming, studies report success rates between 80% and 96% [2,18]. However, ACI is not an option for patients with uncontained OCD lesions with greater than 8 mm of subchondral bone loss or those with bipolar lesions, which are patella defects with an articulating trochlear lesion [10]. The OCA procedure is used for unsalvageable lesions of ≥3 cm^2^ in patients who are not candidates for ACI or as a revision procedure [2,19]. It offers the ability to transfer hyaline cartilage and restore the articular surface with hyaline cartilage without concerns about harvest site morbidity. However, it is limited by graft availability, graft expiration dates, and a difficult learning curve and is ideally suited for patients who are young and active [19].

## Case presentation

**Figure 1:**
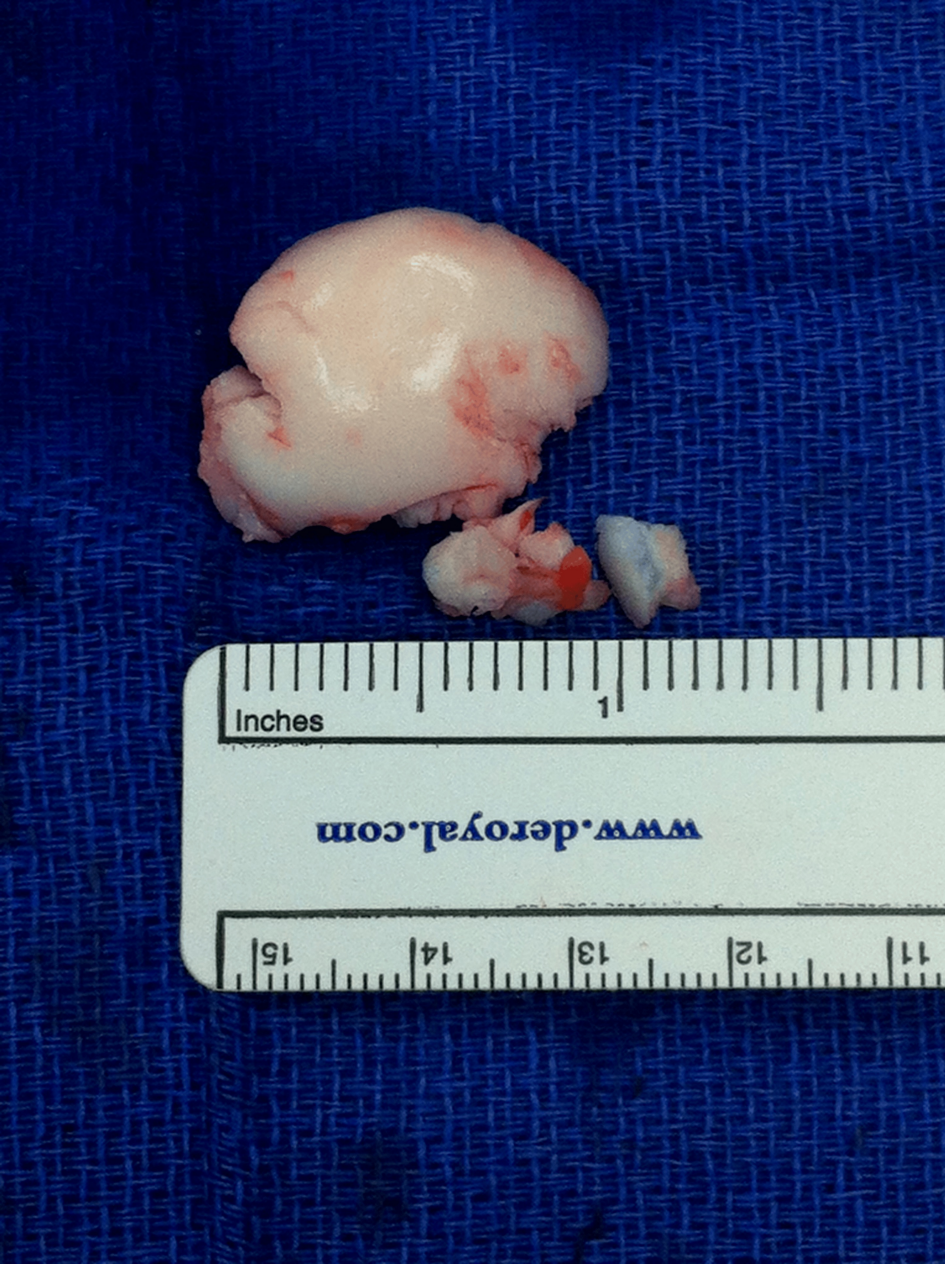
Cartilaginous loose body after surgical removal.

A 13-year-old healthy boy presented for vague right knee pain that worsened with activity. Physical examination was unremarkable, and radiographs revealed possible subchondral irregularities in the lateral femoral condyle. An MRI was ordered and revealed a stable OCD lesion in the weight-bearing portion of the posterior lateral femoral condyle. Initial management included a knee immobilizer and a non-weight-bearing status for six months. The patient then returned to sports with occasional mild discomfort. At age 18, he presented with right knee pain. MRI evaluation revealed a round, 2 cm, cartilaginous loose body overlying the OCD defect, for which the patient underwent a loose body removal and arthroscopic microfracture procedure (Figure 1).

**Figure 2:**
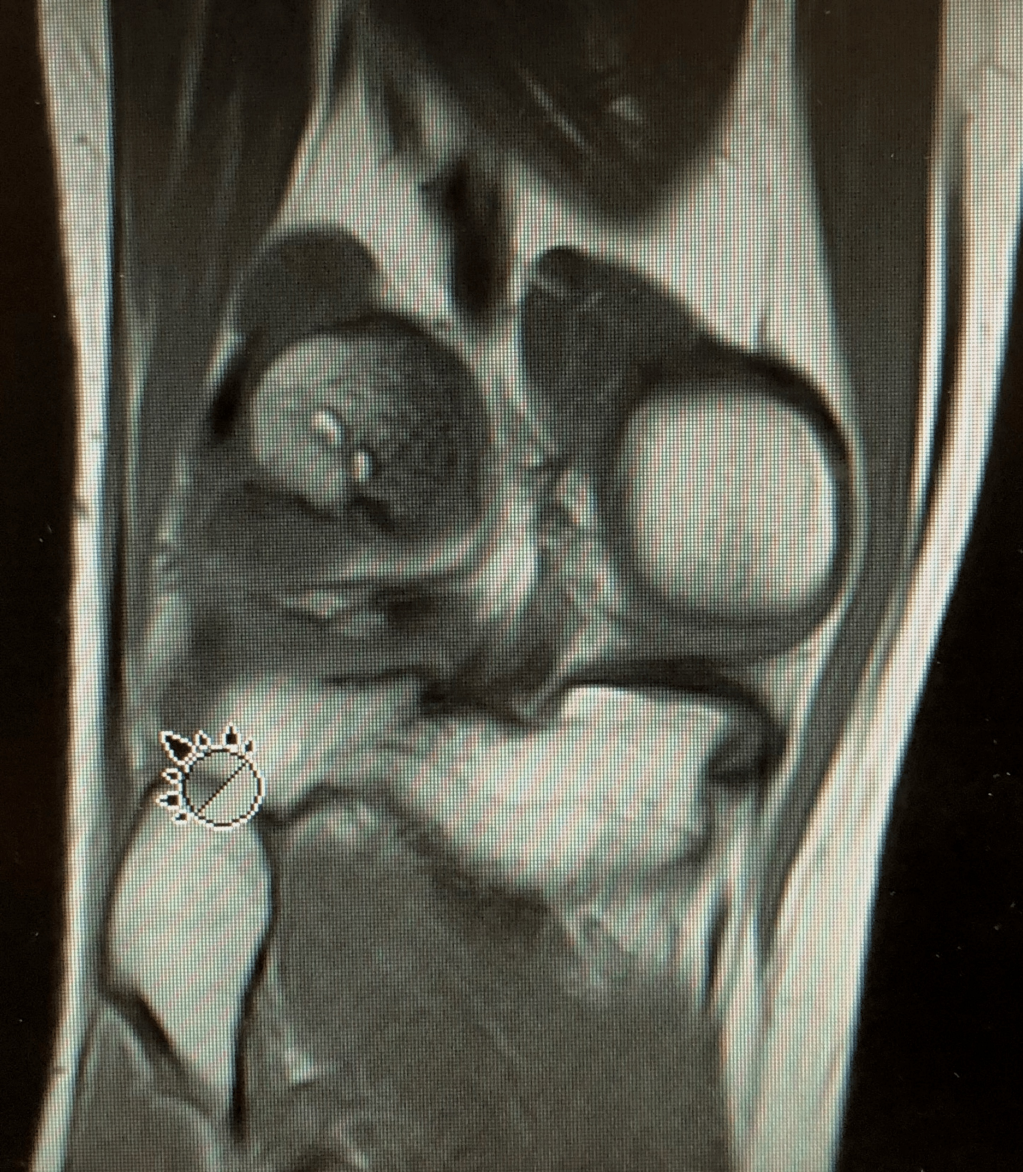
Coronal MRI of the diseased posterior lateral femoral condyle. The cursor is identifying the proximal fibular head for reference. MRI: magnetic resonance imaging

The patient was able to return to physical activity with mild discomfort after activity until age 23, when he presented again for locking and clicking after a valgus knee injury. A subsequent MRI revealed a lateral meniscus tear and that the OCD lesion had become a 1.5 cm by 1.5 cm (2.25 cm^2^) full-thickness defect with adjacent subchondral cysts, edema, and loose chondral fragments (Figure 2).

**Figure 3:**
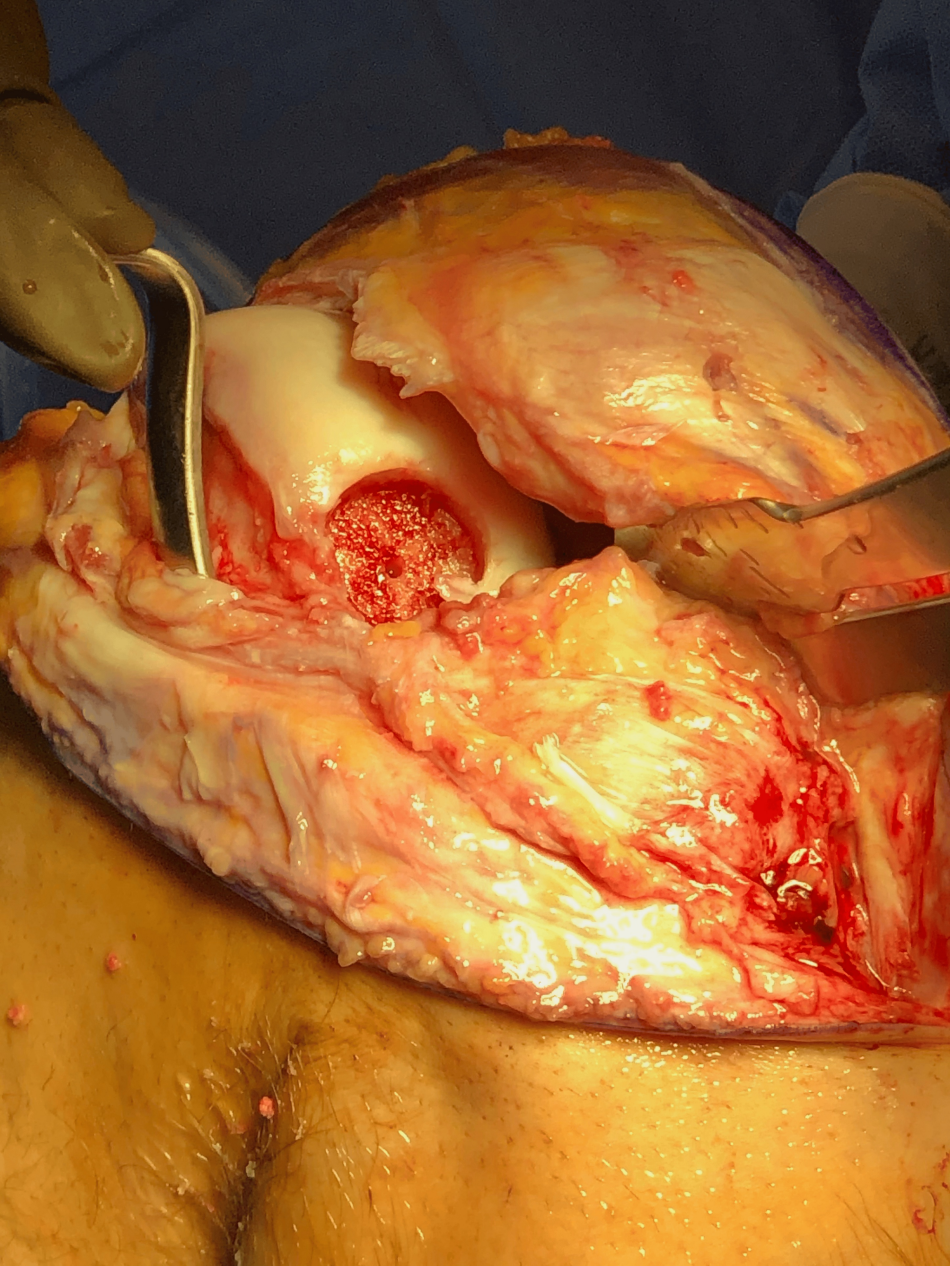
Intraoperative picture of the OCD lesion in the posterior lateral femoral condyle after retracting the patella medially and putting the knee in maximal flexion. Despite its appearance in this figure, the OCD lesion was contained posteriorly. OCD: osteochondritis dissecans

**Figure 4:**
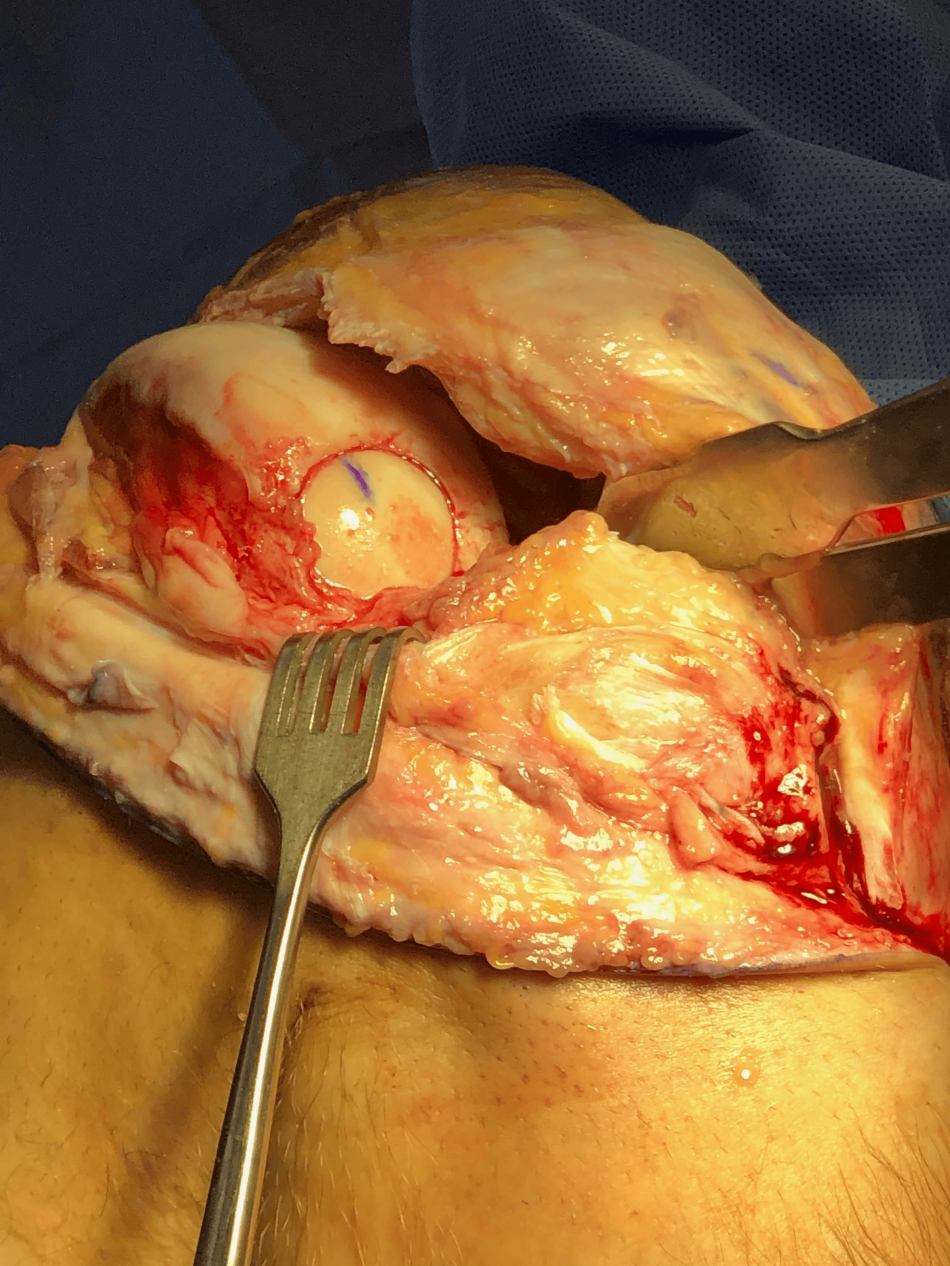
OCD lesion after osteochondral allograft plugging. OCD: osteochondritis dissecans

Due to the lesion’s size, location, and nature, it was considered unsalvageable. Treatment options were discussed, and an osteochondral allograft procedure was recommended. However, the patient was getting married in several weeks, so he opted for an arthroscopic meniscectomy and the debridement of the OCD lesion to avoid postoperative immobilization. After sufficient rehabilitation, he was unable to resume physical activity due to mechanical symptoms and pain, and he consented to undergo an osteochondral allograft procedure. After a donor was found, the patient underwent surgery without complication (Figures 3, 4).

The postoperative course was uneventful, and the patient was able to resume all physical activity. Upon last follow-up, which was approximately five years after surgery, the patient has been able to perform all desired physical activity without symptoms.

## Discussion

This case highlights the complexity of unsalvageable OCD surgical treatment. Due to concerns about being able to harvest enough bone plugs from non-weight-bearing parts of the knee without significant graft site morbidity, an osteochondral allograft procedure was preferred over an OATS procedure. Due to concerns about time, cost, and the lesion’s proximity to the posterior border of the lateral femoral condyle, which nearly made it uncontained, an osteochondral allograft was also preferred over an ACI procedure. This case suggests that the OCA procedure, which is generally indicated for unsalvageable OCD lesions of ≥3 cm^2^, should be considered for smaller, unsalvageable lesions such as this patient’s, which was 2.25 cm^2^, provided that the surgeon has enough experience and comfort performing the procedure [2].

The unusually posterior location of this patient’s OCD lesion in the lateral femoral condyle presented a unique challenge. Simply being able to access the lesion required putting the knee in maximum flexion, which increased the difficulty of the procedure. While all OCA procedures carry the risk of iatrogenic fracture, the proximity of the lesion to the posterior border of the lateral femoral condyle meant that any iatrogenic fracture would have likely been intra-articular, making it even more challenging. Literature predominantly focuses on OCD lesions in the medial femoral condyle, as that is the most common location [3]. However, this case adds to the body of evidence supporting the efficacy of OCA procedures in less common locations, such as the posterior lateral femoral condyle.

## Conclusions

Osteochondral allograft (OCA) procedures are particularly valuable for treating large, unsalvageable osteochondritis dissecans (OCD) lesions, including those in unusual locations. In this case, a 13-year-old boy had an OCD lesion in an atypical location, the posterior lateral femoral condyle, which required operative treatment despite conservative treatment and a microfracture procedure. Due to the location of the unsalvageable OCD lesion, OCA was preferred. OCA is indicated when other treatments, including autologous chondrocyte implantation (ACI) and osteochondral autograft transplantation system (OATS), are not viable. It effectively restores hyaline cartilage without donor site complications, making it suitable for young, active patients. The patient’s successful recovery post OCA underscores its efficacy in restoring joint function and enabling a return to physical activity, even for lesions in less common areas.
